# Supplementary material for: Prevalence of nasopharyngeal bacteria during naturally occurring bovine respiratory disease in commercial stocker cattle
Source: PeerJ. 2025 Jan 20;13:e18858. doi: 10.7717/peerj.18858 (PMC11756368; doi:10.7717/peerj.18858)
Supplement: Supplemental Information 2 — 1Based on rectal temperature and clinical scores, clinically healthy calves were not considered as BRD cases and received no treatment for BRD for the entire study period. 2Based on rectal temperature and clinical scores, calves that were diagnosed as BRD cases received at least 1 antibiotic treatment for BRD. 3Day 0, Day 7, Day 14, and Day 21 denote the day relative to calves’ arrival to the stocker farm. abWithin each column, different superscripts indicate differences (P < 0.05). [file peerj-13-18858-s002.docx]

Table 1: Alpha diversity (Chao1 index and Shannon diversity) at different days in nasopharyngeal samples collected after arrival at the stocker facility.

| Clinically healthy calves^1^ | | | Calves received treatment (at least 1) for BRD^2^ | | |
| --- | --- | --- | --- | --- | --- |
| Sampling day^3^ | Chao1 index | Shannon diversity | Sampling day^3^ | Chao1 index | Shannon diversity |
| Day 0 | 507.08 ± 27.59^a^ | 5.82 ± 0.12^a^ | Day 0 | 473.07 ± 38.90^a^ | 5.76 ± 0.18^a^ |
| Day 7 | 526.72 ± 27.59^a^ | 5.86 ± 0.12^a^ | Day 7 | 458.41 ± 38.90^a^ | 5.54 ± 0.18^a^ |
| Day 14 | 438.58 ± 27.59^a^ | 5.38 ± 0.12^b^ | Day 14 | 495.65 ± 38.91^a^ | 5.79 ± 0.18^a^ |
| Day 21 | 521.10 ± 31.02^a^ | 5.84 ± 0.13^a^ | Day 21 | 393.35 ± 43.17^a^ | 5.16 ± 0.20^a^ |

^1^Based on rectal temperature and clinical scores, clinically healthy calves were not considered as BRD cases and received no treatment for BRD for the entire study period.

^2^Based on rectal temperature and clinical scores, calves that were diagnosed as BRD cases received at least 1 antibiotic treatment for BRD.

^3^Day 0, Day 7, Day 14, and Day 21 denote the day relative to calves’ arrival to the stocker farm.

^ab^Within each column, different superscripts indicate differences (P < 0.05).
