# Supplementary material for: Prevalence of nasopharyngeal bacteria during naturally occurring bovine respiratory disease in commercial stocker cattle
Source: PeerJ. 2025 Jan 20;13:e18858. doi: 10.7717/peerj.18858 (PMC11756368; doi:10.7717/peerj.18858)
Supplement: Supplemental Information 3 — Day 0, Day 7, Day 14, and Day 21 denotes the day relative to calves’ arrival to the stocker farm. abWithin each row, means with unlike letters differ significantly (P < 0.05). [file peerj-13-18858-s003.docx]

Table 2: Temporal changes of the relative abundance of top phyla in NP microbiome of clinically healthy stocker calves (n = 24).

| Phylum | Relative abundance of the bacterial phylum | | | | *P-*value |
| --- | --- | --- | --- | --- | --- |
|  | Day 0 | Day 7 | Day 14 | Day 21 |  |
| *Firmicutes* | 0.67 ± 0.04^b^ | 0.80 ± 0.04^a^ | 0.72 ± 0.04^ab^ | 0.65 ± 0.05^b^ | 0.03 |
| *Proteobacteria* | 0.15 ± 0.03 | 0.10 ± 0.03 | 0.22 ± 0.04 | 0.29 ± 0.04 | 0.13 |
| *Actinobacteriota* | 0.12 ± 0.02^a^ | 0.05 ± 0.02^b^ | 0.04 ± 0.02^b^ | 0.02 ± 0.02^b^ | < 0.0001 |
| *Bacteroidota* | 0.02 ± 0.007^a^ | 0.02 ± 0.007^ab^ | 0.01 ± 0.008^c^ | 0.03 ± 0.008^bc^ | 0.001 |
| *Verrucomicrobiota* | 0.004 ± 0.0007^a^ | 0.003 ± 0.00008^ab^ | 0.002 ± 0.0009^b^ | 0.002 ± 0.0009^b^ | 0.001 |

Day 0, Day 7, Day 14, and Day 21 denotes the day relative to calves’ arrival to the stocker farm.

^ab^Within each row, means with unlike letters differ significantly (*P* < 0.05).
