# Supplementary material for: Prevalence of nasopharyngeal bacteria during naturally occurring bovine respiratory disease in commercial stocker cattle
Source: PeerJ. 2025 Jan 20;13:e18858. doi: 10.7717/peerj.18858 (PMC11756368; doi:10.7717/peerj.18858)
Supplement: Supplemental Information 4 — Day 0, Day 7, Day 14, and Day 21 denotes the day relative to calves’ arrival to the stocker farm. abWithin each row, means with unlike letters differ significantly (P < 0.05). [file peerj-13-18858-s004.docx]

Table 3. Temporal changes of the relative abundance of different genera in NP microbiome of clinically healthy stocker calves (n = 24).

| Phylum | Relative abundance of bacterial genus at different days | | | | *P-*value |
| --- | --- | --- | --- | --- | --- |
|  | Day 0 | Day 7 | Day 14 | Day 21 |  |
| *Mycoplasma* | 0.06 ± 0.05^b^ | 0.30 ± 0.05^a^ | 0.31 ± 0.05^a^ | 0.27 ± 0.06^a^ | 0.01 |
| *Histophilus* | - | - | 0.14 ± 0.04 | 0.19 ± 0.04 | - |
| *Pasteurella* | 0.06 ± 0.02 | 0.02 ± 0.02 | 0.03 ± 0.02 | 0.07 ± 0.02 | 0.41 |
| *Lactobacillus* | 0.06 ± 0.008 | 0.05 ± 0.008 | 0.05 ± 0.008 | 0.04 ± 0.009 | 0.09 |
| *Bacillus* | 0.14 ± 0.01 | 0.11 ± 0.01 | 0.09 ± 0.01 | 0.09 ± 0.02 | 0.30 |

Day 0, Day 7, Day 14, and Day 21 denotes the day relative to calves’ arrival to the stocker farm.

^ab^Within each row, means with unlike letters differ significantly (*P* < 0.05).
