# Supplementary material for: Prevalence of nasopharyngeal bacteria during naturally occurring bovine respiratory disease in commercial stocker cattle
Source: PeerJ. 2025 Jan 20;13:e18858. doi: 10.7717/peerj.18858 (PMC11756368; doi:10.7717/peerj.18858)
Supplement: Supplemental Information 5 — 1NumTrt was defined based on the number of antibiotic treatments they received (0x: never treated; 1x: treated once; 2x: treated 2 times). 2Day 0, Day 7, Day 14, and Day 21 denotes the day relative to calves’ arrival to the stocker farm. abWithin each row, means with unlike letters differ significantly (P < 0.05). [file peerj-13-18858-s005.docx]

Table 4: Relative abundance of top phyla based on the number of antimicrobial treatments (NumTrt) and day in NP microbiome of stocker calves.

| Phylum | NumTrt^1^ | | | | Day^2^ | | | | |
| --- | --- | --- | --- | --- | --- | --- | --- | --- | --- |
|  | 0x | 1x | 2x | *P-*value | Day 0 | Day 7 | Day 14 | Day 21 | *P-*value |
| *Firmicutes* | 0.69 ± 0.03 | 0.72 ± 0.04 | 0.77 ± 0.06 | 0.46 | 0.69 ± 0.05 | 0.83 ± 0.05 | 0.72 ± 0.05 | 0.68 ± 0.05 | 0.05 |
| *Proteobacteria* | 0.21 ± 0.02 | 0.15 ± 0.04 | 0.18 ± 0.05 | 0.36 | 0.15 ± 0.04^bc^ | 0.09 ± 0.04^c^ | 0.21 ± 0.04^ab^ | 0.27 ± 0.04^a^ | 0.03 |
| *Actinobacteriota* | 0.06 ± 0.02^ab^ | 0.09 ± 0.02^a^ | 0.02 ± 0.04^b^ | 0.02 | 0.12 ± 0.03^a^ | 0.05 ± 0.03^b^ | 0.05 ± 0.03^b^ | 0.01 ± 0.02^b^ | < 0.0001 |
| *Bacteroidota* | 0.02 ± 0.006 | 0.03 ± 0.009 | 0.02 ± 0.01 | 0.15 | 0.03 ± 0.01^a^ | 0.01 ± 0.01b | 0.009 ± 0.01^b^ | 0.04 ± 0.01^b^ | 0.002 |
| *Verrucomicrobiota* | 0.003 ± 0.0005 | 0.002 ± 0.0007 | 0.003 ± 0.001 | 0.13 | 0.005 ± 0.0008^a^ | 0.001 ± 0.0008^b^ | 0.002 ± 0.0009^b^ | 0.002 ± 0.0009^b^ | 0.0001 |

^1^NumTrt was defined based on the number of antibiotic treatments they received (**0x**: never treated; **1x**: treated once; **2x**: treated 2 times).

^2^Day 0, Day 7, Day 14, and Day 21 denotes the day relative to calves’ arrival to the stocker farm.

^ab^Within each row, means with unlike letters differ significantly (*P* < 0.05).
