# Supplementary material for: Prevalence of nasopharyngeal bacteria during naturally occurring bovine respiratory disease in commercial stocker cattle
Source: PeerJ. 2025 Jan 20;13:e18858. doi: 10.7717/peerj.18858 (PMC11756368; doi:10.7717/peerj.18858)
Supplement: Supplemental Information 6 — *Numtrt by day effect (presented in Figure 6). 1NumTrt was defined based on the number of antibiotic treatments they received (0x: never treated; 1x: treated once; 2x: treated 2 times). 2Day 0, Day 7, Day 14, and Day 21 denote the day relative to calves’ arrival to the stocker farm. abWithin each row, means with unlike letters differ significantly (P < 0.05) [file peerj-13-18858-s006.docx]

Table 5. Relative abundance of different genera based on the number of antimicrobial treatments (NumTrt) and day in NP microbiome of stocker cattle.

| Genus | NumTrt | | | | Day | | | | |
| --- | --- | --- | --- | --- | --- | --- | --- | --- | --- |
|  | 0x | 1x | 2x | *P*-value | Day 0 | Day 7 | Day 14 | Day 21 | *P*-value |
| *^*^Mycoplasma* | 0.24 ± 0.03 | 0.31 ± 0.04 | 0.31 ± 0.06 | 0.30 | 0.03 ± 0.04^c^ | 0.52 ± 0.04^a^ | 0.32 ± 0.05^b^ | 0.25 ± 0.05^b^ | <0.0001 |
| *Histophilus* | 0.08 ± 0.02 | 0.11 ± 0.03 | 0.12 ± 0.05 | 0.60 | 0.0000024 ± 0.04^b^ | 0.03 ± 0.03^b^ | 0.17 ± 0.04^a^ | 0.22 ± 0.04^a^ | <0.0001 |
| *Pasteurella* | 0.05 ± 0.009^a^ | 0.005 ± 0.01^b^ | 0.006 ± 0.02^ab^ | 0.04 | 0.03 ± 0.02 | 0.01 ± 0.01 | 0.01 ± 0.02 | 0.02 ± 0.02 | 0.34 |
| *Lactobacillus* | 0.05 ± 0.004 | 0.05 ± 0.006 | 0.04 ± 0.008 | 0.06 | 0.07 ± 0.007^a^ | 0.03 ± 0.007^b^ | 0.05 ± 0.007^b^ | 0.04 ± 0.007^b^ | 0.001 |
| *Bacillus* | 0.1133 ± 0.008^a^ | 0.1193 ± 0.01^a^ | 0.1108 ± 0.01^b^ | 0.03 | 0.15 ± 0.02^a^ | 0.07 ± 0.02^b^ | 0.09 ± 0.02^b^ | 0.12 ± 0.02^b^ | 0.007 |

^*^Numtrt by day effect (presented in Figure 6).

^1^NumTrt was defined based on the number of antibiotic treatments they received (**0x**: never treated; **1x**: treated once; **2x**: treated 2 times).

^2^Day 0, Day 7, Day 14, and Day 21 denote the day relative to calves’ arrival to the stocker farm.

^ab^Within each row, means with unlike letters differ significantly (*P* < 0.05)
